# Fine-grained parallelization of fitness functions in bioinformatics optimization problems: gene selection for cancer classification and biclustering of gene expression data

**DOI:** 10.1186/s12859-016-1200-9

**Published:** 2016-08-31

**Authors:** Juan A. Gomez-Pulido, Jose L. Cerrada-Barrios, Sebastian Trinidad-Amado, Jose M. Lanza-Gutierrez, Ramon A. Fernandez-Diaz, Broderick Crawford, Ricardo Soto

**Affiliations:** 1 Department of Technologies of Computers and Communications, University of Extremadura, Polytechnic School, Campus Universitario s/n, Caceres, 10003 Spain; 2 Department of Computer and Aerospace Engineering, University of Leon, Computer Sciences School, Campus de Vegazana s/n, Leon, 24071 Spain; 3 Pontificia Universidad Católica de Valparaíso, Valparaíso, 2362807 Chile; 4 Universidad Central de Chile, Santiago, 8370178 Chile; 5 Universidad Autónoma de Chile, Santiago, 7500138 Chile; 6 Universidad Espíritu Santo, Guayaquil, Ecuador

**Keywords:** Biclustering, Cancer classification, FPGA, Parallelism, Floating-point arithmetic, Metaheuristics, Fitness function

## Abstract

**Background:**

Metaheuristics are widely used to solve large combinatorial optimization problems in bioinformatics because of the huge set of possible solutions. Two representative problems are gene selection for cancer classification and biclustering of gene expression data. In most cases, these metaheuristics, as well as other non-linear techniques, apply a fitness function to each possible solution with a size-limited population, and that step involves higher latencies than other parts of the algorithms, which is the reason why the execution time of the applications will mainly depend on the execution time of the fitness function. In addition, it is usual to find floating-point arithmetic formulations for the fitness functions. This way, a careful parallelization of these functions using the reconfigurable hardware technology will accelerate the computation, specially if they are applied in parallel to several solutions of the population.

**Results:**

A fine-grained parallelization of two floating-point fitness functions of different complexities and features involved in biclustering of gene expression data and gene selection for cancer classification allowed for obtaining higher speedups and power-reduced computation with regard to usual microprocessors.

**Conclusions:**

The results show better performances using reconfigurable hardware technology instead of usual microprocessors, in computing time and power consumption terms, not only because of the parallelization of the arithmetic operations, but also thanks to the concurrent fitness evaluation for several individuals of the population in the metaheuristic. This is a good basis for building accelerated and low-energy solutions for intensive computing scenarios.

**Electronic supplementary material:**

The online version of this article (doi:10.1186/s12859-016-1200-9) contains supplementary material, which is available to authorized users.

## Background

Bioinformatics is an area where we can find many large combinatorial optimization problems [1]. The high size of the space of solutions causes these problems can not be tackled by means of exact searching techniques, which require an excessive computational effort. In these cases, the usual way of obtaining optimal solutions is to consider metaheuristics [2] and particularly Evolutionary Algorithms (EAs) [3]. Nevertheless, even these algorithms can be slow for complex problems, demanding more hardware resources based on current general-purpose processors or Central Processing Units (CPUs). If we identify what part of the algorithm takes more time to be computed, a hardware coprocessor specifically designed to accelerate this function is a direct solution to further speed up the performance. In this sense, the fitness function is a simple but critical operation involved in the metaheuristics. Most of the computing time of the algorithm that solves the optimization problem may be spent running the fitness function, although it could mean a small part of the code.

The core of this work deals with the hardware-level parallelization of the fitness functions used in two bioinformatics problems: gene selection for cancer classification and biclustering of gene expression data. The reason for designing fitness hardware accelerators is twofold. On the one hand, every fitness function is applied to each individual of a population in many bio-inspired metaheuristics; this fact allows us to parallelize the computation of the fitness evaluation phase if we place several copies of the same fitness hardware implementation. On the other hand, fitness functions are usually formulated by means of floating-point arithmetic equations that can involve many operation steps; this way, parallelization of some of these steps using repeated units of the same floating-point operator increases the performance of the design.

**Fig. 1:**
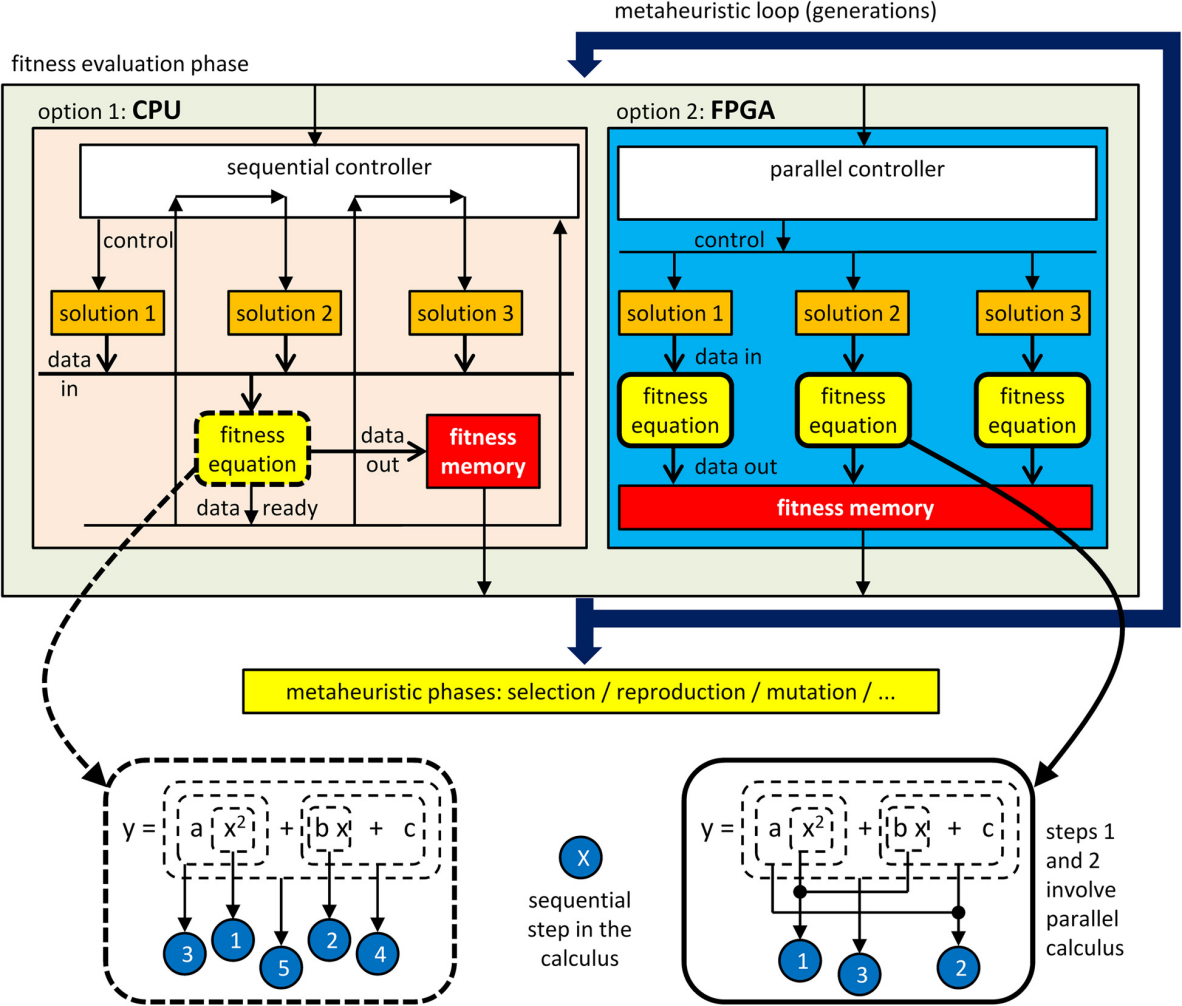
Two possible parallel levels for a FPGA implementation of the fitness phase in a general metaheuristic

Both reasons represent two levels of parallelism: in the bottom, a fine-grained parallelization of the fitness equation; in the top, a fast computation of the fitness evaluation phase applying replicated fitness units in parallel to several individuals of the population. We focused our research mainly on the fine-grained parallelization of the fitness formulation, although on-chip concurrent fitness evaluation has been explored as well. Figure 1 illustrates these considerations, comparing usual CPU sequential programming to custom on-chip parallel systems. We can accelerate the computation of the fitness phase making good use of parallelism: replicated fitness functions working in parallel at the top-level, and parallel computation of the fitness equation at the bottom-level. We can observe that CPU requires sequential steps not only for the evaluation of the fitness of each individual, but for the calculation of the fitness equation.

The hardware implementation of fitness equations is made easier thanks to Hardware Description Languages (HDLs) and Field Programmable Gate Array (FPGA) devices [4]. The FPGA technology favoured the rise of a computing domain that combines software flexibility with hardware performance exploiting the parallel paradigm: Reconfigurable Computing (RC) [5]. This way, a fitness function carefully designed can surpass the CPU performance in similar experimental conditions, as RC has demonstrated in many applications [6]. In addition, we decide on FPGAs instead of other competitive technologies as Graphical Processing Units (GPUs) since FPGAs usually provide better performance and lower power consumption than GPUs [7].

Reconfigurable computing has been successfully applied to many bioinformatics problems, because they have a high parallelism degree. Knowing how to make the most of this parallelism, we can obtain speedups and energy savings needed for intensive computing or real-time applications. In this area, we can find FPGA implementations for DNA matching based on the BLAST algorithm [8], Bowtie short-read mapping [9], epistasis detection [10], molecular modeling [11], and many other algorithms involved in sequence comparison, multiple sequence alignment, RNA and protein secondary structure prediction, gene prediction and phylogenetic tree computation [12], among many others. Nevertheless, these works are usually focused on solving specific problems, dealing with their special characteristics and constraints. Contrary to these approaches, our work tries to get a wide insight into important aspects to take into account when designing accelerators.

This way, our main contribution in this paper is to demonstrate that the fine-grained parallelization of fitness functions based on floating-point arithmetic can surpass the performance given by CPUs, in time and power terms, when they are massively used by metaheuristics for solving large combinatorial optimization problems in bioinformatics. The conclusions of our work can be applied in general to similar cases, because of the representativeness of the fitness functions we have chosen. For this purpose, we have selected two specific fitness functions used in the above mentioned optimization problems by two reasons: on the one hand, there is not enough information about their implementation in FPGAs in the existing literature; on the other hand, they provide different computational workloads and parallelization levels because of their floating-point arithmetic formulations, being representative formulations of other similar functions widely used in bioinformatics.

The hardware implementation of the fitness function can be used as a coprocessor of an embedded CPU running the metaheuristic in the same FPGA. Nevertheless, the need for scalability that large and real-world applications require, and the metaheuristic request of handling many individuals of the population in parallel, make it necessary to consider computing systems consisting of several FPGAs in multicore architectures. This coarse-grained parallelization belongs to the High-Performance Reconfigurable Computing (HPRC), a promising paradigm that exploits the possibilities of FPGAs [13, 14], although it requires to design according to computing models based on specific communication and data-handling techniques [15]. Nevertheless, if we want to develop a computing system based on such large FPGA platforms, the first and mandatory step is to know if the unit to be massively replicated (in our case, the fitness function) is able to give enough speedup with regard to usual CPUs. This is the reason why our research is focused on a worthwhile fine-grained parallelization of the fitness function, since it is the basis for a success scalability that is left as future development.

Summarizing, our proposal presents the performance from a computational perspective. Other performance features closer to the specific bioinformatics problems only can be tackled by the corresponding algorithmic methods and software packages, which are out of the scope of this work.

### Related work

As we pointed out in the previous section, bio-inspired and evolutionary optimization algorithms are very appropriate to be parallelized, not only by applying repeated fitness hardware units in parallel on several individuals of the population, but parallelizing other important parts. For example, the intrinsic parallelism in popular Genetic Algorithms (GAs) [16] allows better speedups. In this line, FPGAs have been successfully applied to parallelize many metaheuristics and optimization algorithms, like Differential Evolution (DE) [17], Particle Swarm Optimization (PSO) [18], Artificial Neural Networks (ANN) [19], and Ant Colony Optimization (ACO) [20], among many others.

The high performance cost of the fitness evaluation phase in relation to the overall computing time of the metaheuristic is a well-studied fact in the literature. Fitness evaluation can take up to 95 % of the total execution time in genetic programming [21] or 64 % in GAs with evolutionary mapping [22]. In general, many works have demonstrated that the execution time of the applications will mainly depend on the execution time of the fitness function [23, 24].

The above considerations move us to implement the fitness functions in hardware to enhance the system performance. These functions have been accelerated by means of FPGA devices in genetic programs for financial markets [25], spatial image filters [26], filtered image signals [27], test cases [28], and many other engineering applications.

The first bioinformatics problem in our study is gene selection for classification of high dimensional Microarray data in cancer disease. This optimization problem has been studied using mainly GAs and Support Vector Machines (SVMs), where the GA is used to evolve gene subsets whose fitness is evaluated by a SVM classifier. In this line, there are approaches based on single objective [29] and multi-objective [30] points of view. Nevertheless, we have not found any FPGA implementation of fitness functions associated to this problem. Therefore, we offer novel insight into its hardware parallelization.

The second optimization problem considered in our research deals with biclustering of gene expression data, which has been tackled by means of custom evolutionary algorithms [31, 32]. We have not found any FPGA implementation of the fitness function as it is formulated in these works. Nevertheless, FPGAs have been applied in a related work, in order to accelerate the Geometric Biclustering (GBC) algorithm [33]; in this work, we compared the FPGA implementation with multi-core CPU and GPU architectures, and found out that FPGA achieved higher speedup for large microarrays, as well as lower power consumption.

### Two case studies in bioinformatics

We have tackled the implementation of the two above mentioned bioinformatics problems following the same strategy: first, we design a fine-grain parallel circuit that implements the fitness function; then, we measure the speed-up with regard to current general-purpose processors for just one fitness evaluation; finally, we estimate the performance when several fitness circuits evaluate individuals in parallel, taking into account the area constraints for a single FPGA. This approach allows us to apply the fitness circuits as coprocessors of an embedded processor that drives different optimization algorithms. This methodology is similar to other studies, as [34, 35], where a single FPGA contains multiple instances of fitness circuits to evaluate possible solutions in parallel, together with the optimization algorithm driven by an embedded processor.

There are many other bioinformatics problems involving metaheuristics with fitness functions similar to these two cases, specifically with regard to the floating-point arithmetic [36, 37]. This way, analyzing the FPGA implementation of the two case studies can contribute to expect good computing speedups in other works.

#### Gene selection for cancer classification

The analysis of microarray-based gene expression allows us to compare between the gene expression levels of cancerous and normal cells, in order to select the genes under suspicion [38]. These genes are useful for cancer classification, but hard to be selected when the number of genes (*M*) and samples (*N*) are very high, shaping a combinatorial optimization problem.

A common approach to face this challenge consists in selecting a subset of suspicious genes for cancer classification. This is the basis of many metaheuristics where the individuals of the population are gene subsets. We have considered a fitness function given by (1), where *x* is the subset, *A*(*x*) is the leave-one-out-cross-validation accuracy provided by a classifier, *R*(*x*) is the number of selected genes in the subset, and *w*_1_ and *w*_2_ are weights for the accuracy level and the number of selected genes, respectively [30]. This fitness function must be maximised by the metaheuristics in order to find an optimal gene subset.

1 $$ F(x) = w_{1} A(x) + w_{2} \frac{M - R(x)}{M}  $$

The top-level circuit to test the fitness function (Additional file 1: Figure S1) is composed of *NF* instances of the fitness circuit, *NC* instances of a floating-point comparator, and a controller that drives and parallelizes the operations involved in *F*. The value of *NF* depends on the FPGA area.

The mission of the controller is to handle the different steps of the test process, which follows this scheme:

1. The controller simultaneously sends different subsets to the fitness units, together with a start instruction and other values involved in the fitness calculation.
2. The fitness units start to compute *F* in parallel for each subset. The calculation in each unit is parallel too.
3. Once all the *NF* units have calculated the fitness values, they are sent in parallel by pairs to *NC*=*NF*/2 floating-point comparators.
4. The comparators determine the highest values of the fitness pairs. Once all the comparisons have finished, the best values are compared again by pairs, this time by means of *NC*/2 comparators.
5. The comparison process continues up to reach the last pair of higher values, where the highest one is given back to the controller.

The fitness circuit implements the arithmetic operations involved in *F*, some of them in parallel. The architecture of the fitness unit (Additional file 1: Figure S2) is composed of several arithmetic modules and a fitness controller. The fitness controller drives the arithmetic operations according to (1), where three operations are performed in parallel: *w*_1_*A*(*x*), *w*_2_/*M* and *M*−*R*(*x*). This architecture needs three floating-point arithmetic operators (adder, multiplier and divider) and an integer to float converter. The fitness controller supplies the operands to the arithmetic modules and receives the results. Once the calculation of *F* has been completed, the fitness controller gives it back to the controller.

#### Biclustering of gene expression data

This problem deals with numerical matrices that represent information extracted from microarray data. These matrices can be built using clustering or biclustering methods [39]. Clustering methods gather together genes with a similar behaviour under all the experimental conditions, using algorithms based on genes similarity, whereas biclustering methods find subsets of genes with the same behaviour under a subset of experimental conditions.

A general bicluster is represented by a matrix *B* of *I* rows (number of experimental conditions) and *J* columns (number of genes), where the element *b*_*ij*_ is the expression level of the gen *j* under the experimental condition *i*.

Since biclustering is more complex than clustering, several evolutionary algorithms have been applied in order to find biclusters. These algorithms consider as fitness function a measure for assessing the quality of biclusters. One usual measure is the Mean Squared Residue (MSR), that provides lower values for better biclusters. The MSR value is calculated following these steps:

1. Calculation of the means *biJ* of each row *i* (2).
2. Calculation of the means *bIj* of each column *j* (3).
3. Calculation of the mean *bIJ* of the entire matrix (4).
4. Calculation of the residue *r*_*ij*_ of each matrix element (5).
5. Calculation of the MSR (6).

2 $$ b_{iJ}[i]=\frac{\sum\limits_{j=0}^{J-1}b_{ij}}{J}=\frac{\mathit{sum\_biJ}_{i}}{J}  $$

3 $$ b_{Ij}[j]=\frac{\sum\limits_{i=0}^{I-1}b_{ij}}{I}=\frac{\mathit{sum\_bIj}_{j}}{I}  $$

4 $$ bIJ=\frac{\sum\limits_{i=0}^{I-1}\sum\limits_{j=0}^{J-1}b_{ij}}{J}=\frac{\mathit{sum\_bIJ}}{I \cdot J}  $$

5 $$ r_{ij}=b_{ij}-\mathit{biJ}_{i}-\mathit{bIj}_{j}+\mathit{bIJ}  $$

6 $$ MSR=\frac{\sum\limits_{i=0}^{I-1}\sum\limits_{j=0}^{J-1}(r_{ij})^{2}}{I \cdot J}  $$

This procedure is highly parallelizable. There are different ways to parallelize the calculation of MSR, according to the experimental constraints: the more resources we have, the more parallelization we can achieve. Since the parallelism comes basically from the use of replicated circuits of the floating-point arithmetic operators, the FPGA device can host different number of these units depending on two factors: the specific FPGA device (family and model) and the size of the bicluster. Due to this reason, we have considered two different parallelization models to compute MSR.

**Fig. 2:**
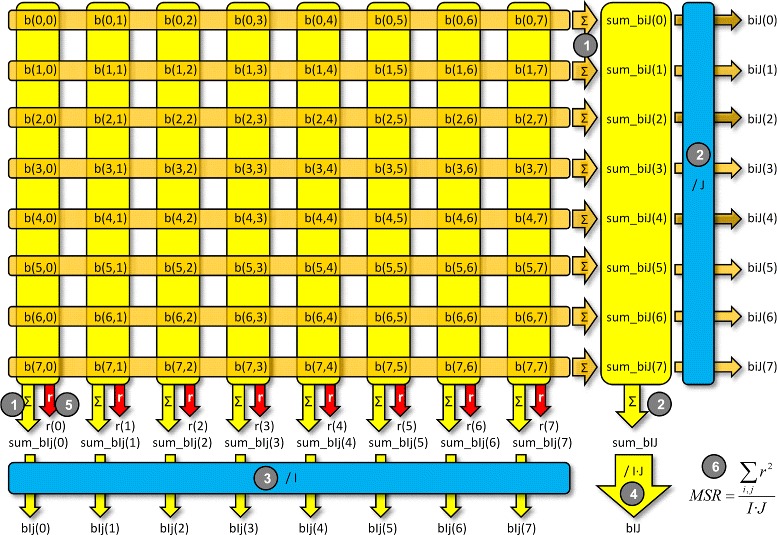
Partially-parallel MSR computation for a 8 ×8 bicluster

We name the first model as *MSR partially parallelized*. This is a procedure useful for bigger matrices or FPGA devices with lower area, where we can only use a limited number of repeated circuits for the arithmetic operators. This procedure involves more sequential steps than in the case where we have as many multipliers as elements *b*_*ij*_ in the matrix. This way, the computation of MSR follows six sequential steps, each of them composed of parallel tasks, as Fig. 2 shows an example of a 8 ×8 bicluster:

1. The elements *b*_*ij*_ of each row and column are added in parallel, obtaining at the same time the values of *sum*_*biJ*_*i*_ and *sum*_*bIj*_*j*_.
2. The sum *sum*_*bIJ* of all the elements *b*_*ij*_ of the matrix (adding the values of *sum*_*biJ* for all the rows) is obtained in parallel together with the values of *biJ*_*i*_ (obtained dividing *sum*_*biJ*_*i*_ by *J*) according to (2).
3. The values of *bIj*_*j*_ are obtained in parallel dividing the corresponding *sum*_*bIj*_*j*_ by *I*, according to (3).
4. The value of *bIJ*, according to (4), is calculated dividing *sum*_*bIJ* by *I*·*J*.
5. The values of *r*_*ij*_, according to (5), are calculated in parallel by rows, but sequentially by columns, taking into account that the number of parallel floating-point multipliers is limited.
6. Finally, the value of MSR, according to (6), is obtained parallelizing the calculation of *r*^2^.

**Fig. 3:**
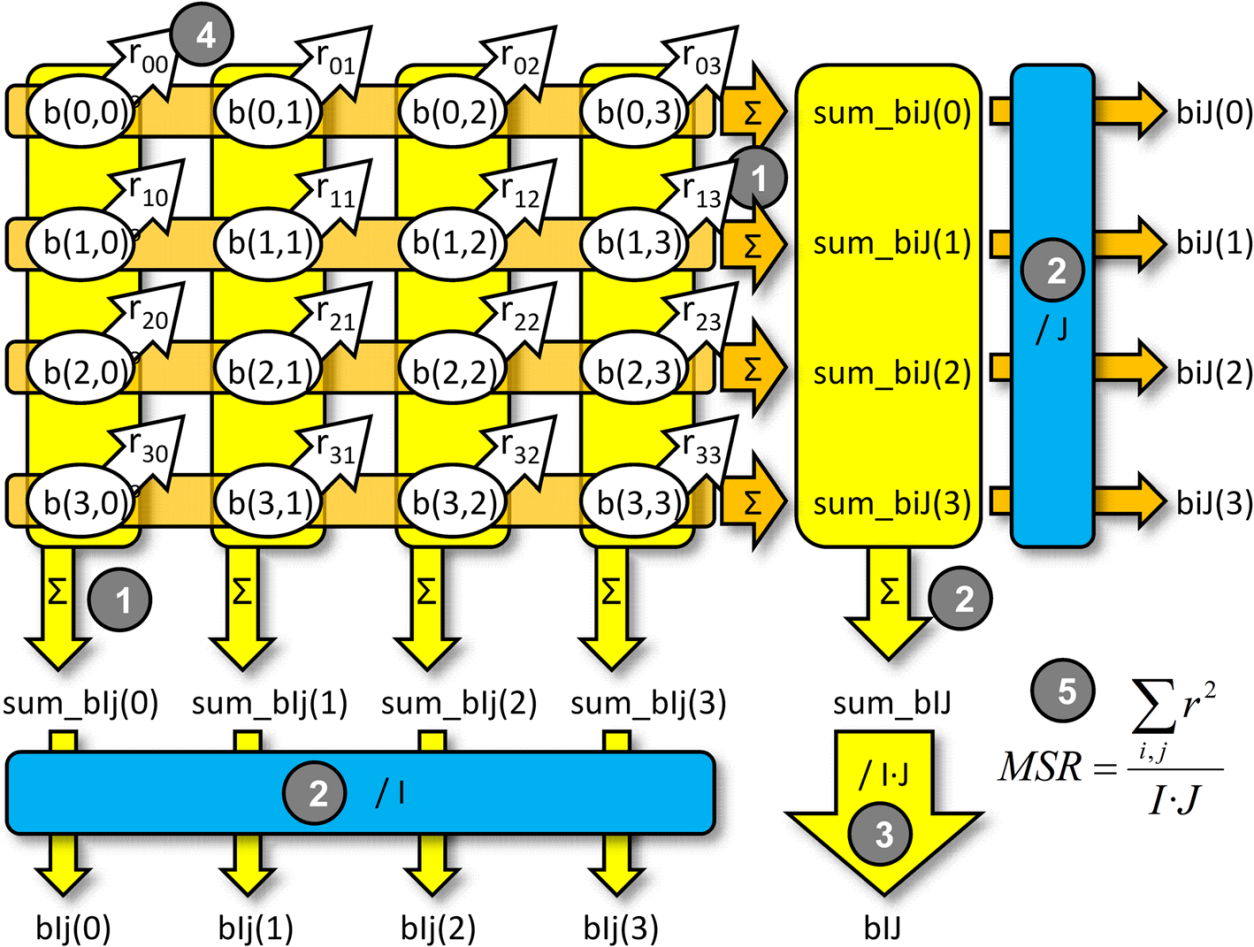
Fully-parallel MSR computation for a 4 ×4 bicluster

The *MSR fully parallelized* model parallelizes the MSR computation in a higher grade. This procedure can be applied to large FPGA devices or smaller matrices. In this case, the MSR calculation follows five sequential steps, each of them also composed of parallel tasks, as Fig. 3 shows for an example of a 4 ×4 bicluster:

1. The first step is the same as in the *MSR partially parallelized* model: calculation of *sum*_*biJ*_*i*_ and *sum*_*bIj*_*j*_.
2. Now we increase the parallelism with regard to the first model, calculating in parallel *sum*_*bIJ*, *biJ*_*i*_ and *bIj*_*j*_.
3. This step corresponds with the fourth step in the first model: calculation of *bIJ*.
4. Now we can calculate *r*_*ij*_ in a fully parallel way, because we have more parallel floating-point multipliers.
5. The last step calculates MSR as the previous model does.

The top-level circuit that measures the MSR performance (Additional file 1: Figure S3), just like the fitness function for the first bionformatics problem, is composed of *NF* instances of the fitness circuit, *NC* instances of a floating-point comparator, and a controller. The value of *NF* and the corresponding *NC*=*NF*/2 also depend on the FPGA area.

The controller and the fitness circuits have different implementations according to the parallelization model and the bicluster size. The implementation version is identified by one letter (*f* for the partially parallelized model, and *a* for the fully parallelized one) followed by the matrix size. In addition, the number of fitness and comparator units is specified for the controller. For example, *controller-f16x8-NF6-NC3* denotes the circuit implementation for a bicluster of 16 experimental conditions and 8 genes driven by the partially parallelized model using 6 parallel fitness units; in this case, the fitness circuit associated with this controller is identified as *msr-f16x8*.

The architecture of the fitness circuit (Additional file 1: Figure S4) may contain different number of adders, multipliers, dividers and integer-to-floating point converters, according to the implementation version. Each implementation version takes into account specific parallelization and resource use. For example, the design *controller-f8x8-NF4-NC2* hosts 2 comparators and 4 fitness units of type *msr-f8x8*, each of them containing 8 adders, 8 multipliers, 8 dividers and 8 converters, whereas a *controller-a4x4-NF4-NC2* circuit counts the same number of comparators and fitness units of type *msr-a4x4*, each of them containing 16 adders, 16 multipliers, 16 dividers and 16 converters. Obviously, the designs following the fully-parallelized model need much more hardware resources than the partially-parallelized model, even for smaller biclusters.

## Results

This section summarizes the tools, hardware resources, and implementation keys from which the results were obtained.

### Design tools

We have designed the fitness units, controllers and assistant circuits using programming languages and tools specifically used for designing with reconfigurable hardware. The main cores were programmed using VHDL hardware description language [40]. This is a is very efficient and known language, specially when we are programming at the register-transfer level, allowing to program algorithms abstracting away the hardware as far as it is possible.

On the other hand, we have used Xilinx ISE 14 software suite [41] for the simulation, synthesis and implementation of the top-level circuits. This suite contains two important tools: on the one hand, CORE Generator System tool was used for generating the circuits for the floating-point arithmetic operators; on the other hand, ISim simulator was used for testing the top level circuit and measuring the time responses, very useful to calculate the speedups of the FPGAs with regard to CPUs.

The design methodology follows some steps, starting from the programming of the circuits using VHDL and CORE Generator tool. In this step is mandatory to do the maximum parallelization effort in order to design an efficient architecture. Once built the codes, the synthesis and implementation step allows obtaining the minimum clock frequency for a determined FPGA device. Using this information, a VHDL testbench customized with the corresponding clock period can simulate the top level design using ISim, obtaining the time response of the circuit, which will be used to calculate the FPGA speedup.

### Hardware resources

Table 1 shows the hardware used for the experiments: FPGA devices for implementing the fitness circuits and general-purpose CPUs for comparing the performance results.

**Table 1:** Hardware resources

Devices	Features			
FPGAs:	Technology	Logic cells	DSP slices	RAM blocks
xc5vlx330-1ff1760	65nm	331,776	192	10,368 kB
xc6vlx550t-2ff1759	40nm	549,888	864	22,752 kB
xc6slx150-3fgg676	45nm	147,443	180	4,824 kB
CPUs:	Technology	GHz		
Core2-E6750	65nm	2.6		
i7-950	45nm	3.07		
i5-2430	32nm	2.4		
i7-2600	32nm	3.4		

The selected Xilinx FPGA devices offer a representative range of features, including the low-cost Spartan6 (xc6slx150), the high-performance Virtex6 (xc6vlx550t) and the balanced Virtex5 (xc5vlx330). These devices may be characterized by four important features that describe the process technology (Complementary Metal-Oxide-Semiconductor -CMOS- depth in nanometers), the number of logic cells (as indicator of the area available to host the circuits), the number of internal Digital Signal Processor (DSP) slices (related to the speed of the floating-point arithmetic operators) and the number of memory blocks (useful to handle the circuit data).

We measured the performance of our three FPGA devices with the post-placement and routing simulation tool provided by the implementation environment. The validation of the results consisted in comparing the simulation times of the Virtex5 device with those measured with custom circuits on a prototyping board that hosted the xc5vlx330 device: Xilinx University Program Virtex5 Development Kit. Since both times were almost equal, we can approve the simulation results corresponding to the other FPGA devices.

In order to establish a valid FPGA vs CPU comparison, and properly analyze the performances, we should consider the use of contemporary devices with similar technologies. This reason led us to use several processors of different CMOS technologies and clock frequencies, as we can see in Table 1.

### Implementation keys

We designed custom circuits to test the performance of the fitness function instead of using embedded processors because these ones take up an area that, otherwise, would be useful for hosting more parallel fitness circuits.

Each synthesis was repeated several times following different strategies in order to obtain the highest clock frequency. On the one hand, we considered three optimization synthesis profiles: default, timing performance with physical synthesis, and timing performance without input/output blocks packing; other synthesis profiles were discarded because of their worse results. On the other hand, we have tested two possibilities when it comes to synthesizing the floating-point arithmetic operators by CORE Generator: using internal DSPs or logic blocks in the architecture optimization. If we consider DSPs, the performance can be better, but the limited number of DSPs forces us to consider digital logic if we want to have more parallel units, involving more area consumption; this tradeoff between number and performance of parallel operators must be evaluated in each case.

This way, each design was synthesized up to 6 times (according to the 3 synthesis profiles and the 2 possibilities of using DSPs in the operator circuits), recording the best result among the obtained ones. For example, for the fitness function in the gene selection for cancer classification problem, we tested 6 cases (8, 16, 32, 64, 128 and 256 parallel fitness units); therefore, 6 cases x 3 synthesis profiles x 2 operator optimizations = 36 synthesis experiments were performed. Depending on the *NF* value considered, the synthesis took from 1 hour to 2 days, also according to the processor used among those listed in Table 1. This means many days running synthesis processes. For the fitness function in the biclustering of gene expression data problem, 8 cases corresponding to different matrix sizes and parallelizing strategies were tested (f4x4, f8x8, f16x8, f16x16, f30x50, f32x64, a4x4, a5x5), totalizing 48 syntheses.

Each synthesis reports interesting data with regard to the scalability and performance of the fitness circuits:

1. Area occupation. Several indicators (slice registers, slice Look-Up-Tables and occupied slices) allow us to calculate the number of circuits that we can replicate in the same FPGA device in order to work in parallel. Depending on the values returned by these indicators and the FPGA family and model, a different number of such circuits can be considered.
2. Timing performance. The value of the maximum frequency (MHz) (that corresponds to the minimum clock period in nanoseconds) allows us to determine the time to process the fitness function; if we consider *NF* parallel units of the fitness circuit, the time to process the different solutions is equal to that time.
3. Power consumption. Nowadays, it is very important to design energy-aware circuits in order to minimize operation costs when solving problems that involve massive computations along the time. The synthesis process tells us the power (watts) consumed by the fitness circuits.

## Discussion

Among the many data returned by the synthesis processes, we analyze mainly the timing reports, since they provide the speedup of FPGA versus CPU (of course, we have checked the numerical results are the same in both FPGA and CPU implementations). We understand by timing performance the reciprocal of the computing time *T* [42]. To compare the performance of FPGAs and processors, we say that the speedup of FPGA versus CPU is *T*_*CPU*_/*T*_*FPGA*_. Hence, a speedup greater than one means that FPGA is faster than CPU; otherwise the processor wins. It is important to realize that both values, *T*_*CPU*_ and *T*_*FPGA*_, measure the same number of fitness evaluations; in the first case, using a loop of sequential computations, whereas the second case considers a parallel computation of *NF* fitness circuits.

**Fig. 4:**
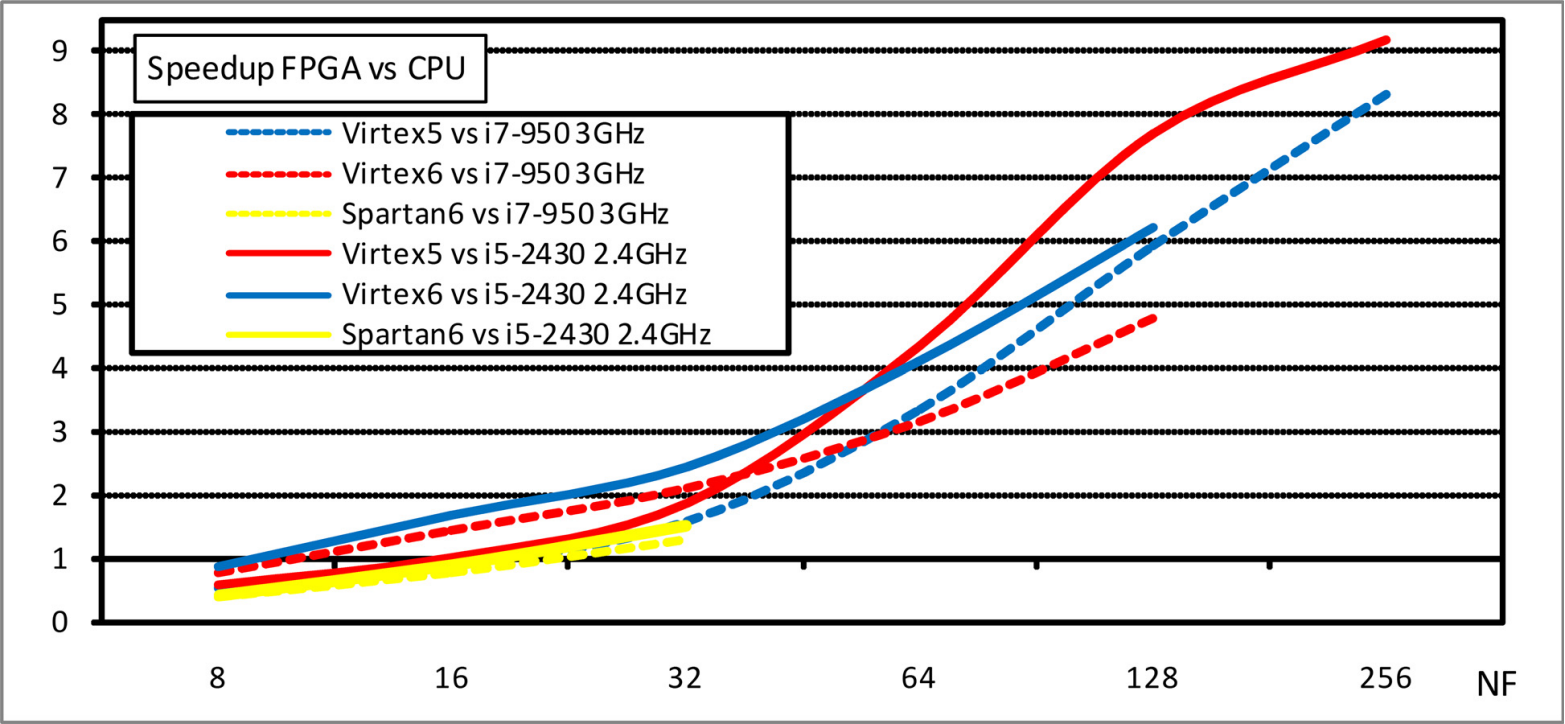
Speedup FPGA vs CPU for the fitness function in the gene selection for cancer classification problem

According to this speedup definition, and taking into account the maximum number of parallel fitness circuits that can operate in parallel in the same FPGA, Fig. 4 shows that FPGAs are much faster than CPUs computing the fitness phase in the gene selection for the cancer classification problem, according to the different FPGA devices, two processors, and a wide range of values for *NF*. The FPGAs provide better speedups than CPUs (up to x9), even for the highest performance processor. We can observe that, the more parallel fitness units we consider, the better speedup we obtain, although this increase is not linear, because of the more dense top level circuits that slow down the clock frequency. In addition, Virtex5 provides better performance than Virtex6 because of the memory constraints to handle the synthesis of large designs (this constraint impedes to consider 256 fitness circuits for the Virtex6 device). Finally, since the Spartan6 device is a low-cost FPGA, it provides much lesser area than the other devices, making it impossible to host more than 32 parallel fitness units.

**Fig. 5:**
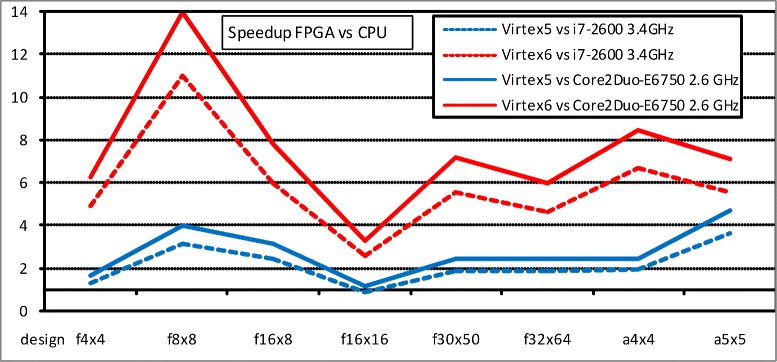
Speedup FPGA vs CPU for the fitness function in the biclustering of gene expression data problem

A similar analysis can be done seeing Fig. 5, that shows the speedups in the biclustering of gene expression data problem for experiments that use different matrix sizes and parallelizing strategies. Here, we have considered the high and medium-performance FPGA devices and other two different CPUs. Now, we obtain higher speedups than in the former bioinformatics problem (up to x14), and for all the cases, because of the higher parallelization degree in both, the fitness equations and the matrix operations. In addition, we can extract two interesting conclusions. On the one hand, the *MSR fully parallelized* model provides better performance than the *MSR partially parallelized* model for equal bicluster sizes, as the first one involves more parallel operations. Nevertheless, the highest number of replicated floating-point arithmetic operators runs out first the FPGA area available: this is the reason why we can not consider large matrix sizes in the fully parallelized model. On the other hand, when using the *MSR partially parallelized* model, since it parallelizes mainly by rows, we should compare matrix sizes with the same number of rows, for example f16x8 with f16x16. In this case, we find that the performance is better with fewer columns, as the lower number of floating-point arithmetic operators allows more area to host more fitness units working in parallel, which has more weight in the performance than the bicluster size.

The speedups for the second bioinformatics problem (biclustering) are good in all the cases and higher than for the first problem (gene selection). We find the reason mainly in the parallelism degree of the fitness circuit design, rather than in the number of such circuits working in parallel. The bottom level of the fine-grained parallelization is the fitness circuit, which is composed of some basic floating point operators: adders, dividers, multipliers and integer to float converters. This way, the more floating point operators running in parallel, the better performance we expect. We find 4 operators in the fitness circuit for gene selection, whereas the fitness implementations for the different bicluster sizes and architectures go from 8 to 32 operators. The number of floating-point operators running in parallel has great influence on the final performance, even more than the number of replicated fitness circuits. In fact, the number of parallel units is higher in the first problem: the performance speedup for the gene selection test with 256 fitness units is x9, whereas 20 units in a f8x8 bicluster gives x14. The reason is simple: a greater number of parallel fitness units in the same FPGA device implies more circuit density in the top level architecture (more communication buses, interconnection blocks, logic cells, etc.), which produces smaller clock frequencies with the corresponding time response decrease.

**Fig. 6:**
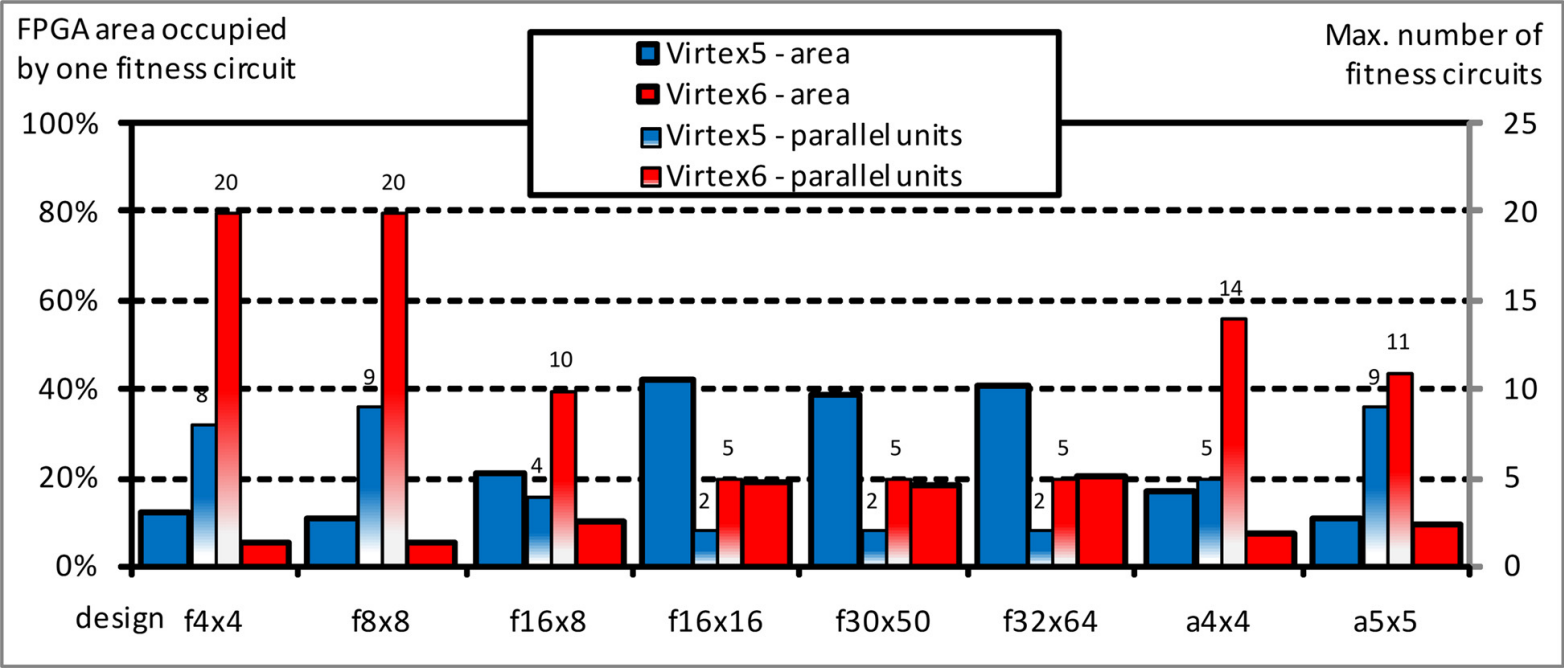
FPGA area occupied by just one fitness circuit and maximum number of such units to work in parallel in the biclustering problem

The ratio of the area occupied by just one fitness circuit to the maximum number of such circuits that the FPGA can host can be seen in Fig. 6, for the second bioinformatics problem: we can have more fitness units in larger FPGAs or considering designs that use lower slice resources. Summarizing, there is a strong relationship between the area required to implement a single fitness function and the bicluster size. Furthermore, increasing the area required for the fitness function decreases the total number of parallel units that can be implemented on FPGA. Therefore, it is needed to establish a tradeoff for each experimental framework.

Finally, it is interesting to know the power consumption of the fitness circuits, since they have an important impact in the metaheuristics as we saw in the related work section. This impact involves high energy when the optimization problems demand intensive computations along the time.

The power consumption in the FPGA is calculated by the XPower Analyzer tool inside the Place&Route phase of the implementation, and the CPU energy is measured by the Powerstat tool under Linux, using the Advanced Configuration and Power Interface (ACPI) battery data of a laptop. Considering the gene selection for cancer classification problem with *NF* =8, we obtained a power consumptions of 3, 6.4 and 0.2 watts for Virtex5, Virtex6 and Spartan6 devices respectively, whereas the Core2-E6750 processor used around 40 watts for the same configuration. This means that the FPGA reduces the power consumption at least 84 % with regard to the CPU.

## Conclusions

The interest of applying the reconfigurable computing technology based on FPGAs to implement the fitness function lies in the possibility of accelerating the evaluation phase in many metaheuristics. This phase evaluates a population of solutions to a combinatorial optimization problem in the bioinformatics domain. The design of a custom circuit that implements the fitness equation allows its replication in several processing units that work in parallel and, thus, accelerate the evaluation phase.

Since many optimization problems in bioinformatics define fitness functions as floating-point arithmetic operations, we have tested two of them in order to check specific implementation features: area occupation, response time and energy, mainly. From these values we can obtain the number of replicated units working in parallel and the time for the evaluation phase. The results show that FPGAs provide better performances than CPUs, not only because of the parallelization of the arithmetic operations of the fitness, but also thanks to the concurrent fitness evaluation for several individuals of the population in the metaheuristic.

Finally, the very low power consumption of the FPGA devices in comparison to CPUs proves that FPGA-based parallel computing environments are excellent low-cost computing solutions for intensive computing scenarios.

As future research line, we will tackle the connection of these accelerated fitness functions with evolutionary frameworks for solving the combinatorial optimization problems. The main idea is to implement an EA in software, leaving the intensive fitness computation to the hardware.

## Methods

The methodology for designing and simulating the different circuits considers the software tools described before in Section “Design tools”. Assuming that these tools require depth knowledge in hardware description languages, as well as the corresponding software licenses from the vendors, the general methodology followed in this work is composed of nine steps:

1. Build a hardware project under Xilinx ISE 14.6 environment, selecting the corresponding FPGA device.
2. Design the code files corresponding to the bioinformatics problem: VHDL files. This is the core step of the work, meaning the greatest effort of the project.
3. Generate the floating-point arithmetic operators from the CoreGen tool.
4. Synthesize and implement the design, activating the corresponding option to obtain advanced reports.
5. After the implementation phase, check the clock period required.
6. Simulate the design using a VHDL testbench, adjusting the clock period to the reported before.
7. Check the time response for the FPGA.
8. Build a C code to run the fitness function in usual microprocessors, compile, run and check the time response.
9. Compare the measured time against the obtained in the FPGA, and calculate the speedup.

## Additional file

Additional file 1 This document includes Figures S1, S2, S3 and S4 with detailed views of the top-level and fitness circuits. (PDF 825 kb)

## Abbreviations

ACO: Ant colony optimization
ACPI: Advanced configuration and power interface
ANN: Artificial neural networks
CMOS: Complementary metal-oxide-semiconductor
CPU: Central processing unit
DE: Differential evolution
DSP: Digital signal processor
EA: Evolutionary algorithm
FPGA: Field programmable gate array
GA: Genetic algorithm
GBC: Geometric biclustering
GPU: Graphical processing unit
HDL: Hardware description language
HPRC: High-performance reconfigurable computing
MSR: Mean squared residue
PSO: Particle swarm optimization
RC: Reconfigurable computing
SVM: Support vector machine
