# Supplementary material for: Fine-grained parallelization of fitness functions in bioinformatics optimization problems: gene selection for cancer classification and biclustering of gene expression data
Source: BMC Bioinformatics. 2016 Aug 31;17(1):330. doi: 10.1186/s12859-016-1200-9 (PMC5007680; doi:10.1186/s12859-016-1200-9)
Supplement: Additional file 1 — This document includes Figures S1, S2, S3 and S4 with detailed views of the top-level and fitness circuits. (PDF 825 kb) [file 12859_2016_1200_MOESM1_ESM.pdf]

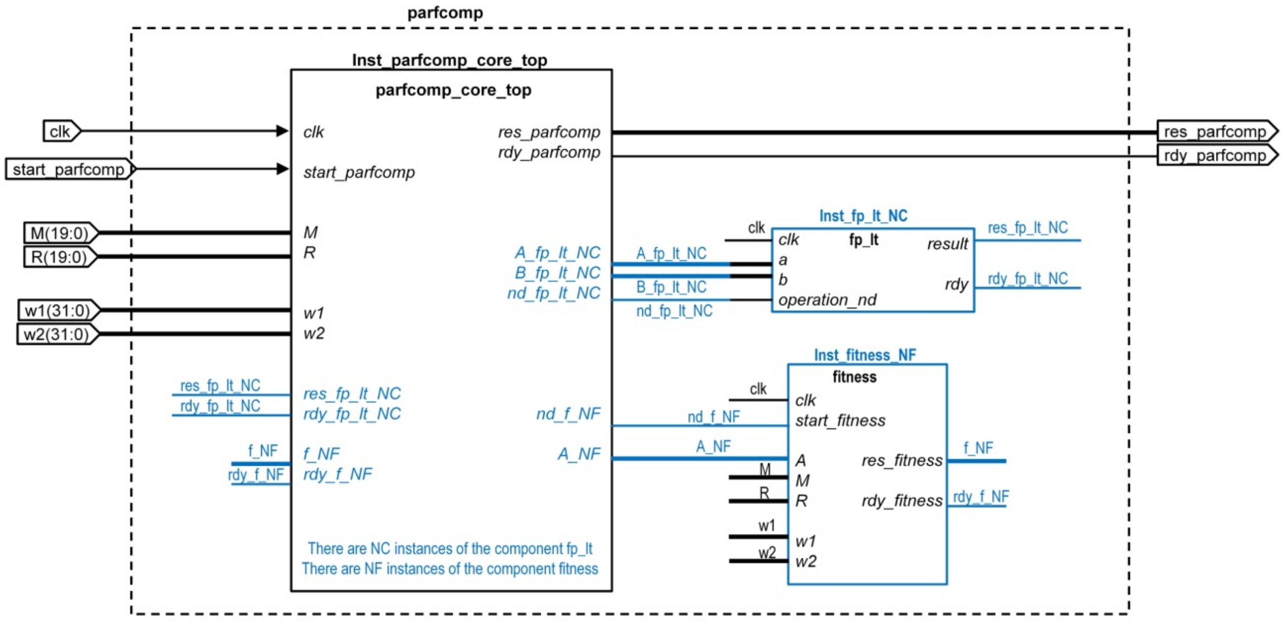

Figure S1. Controller for the parallel fitness computations and comparisons.

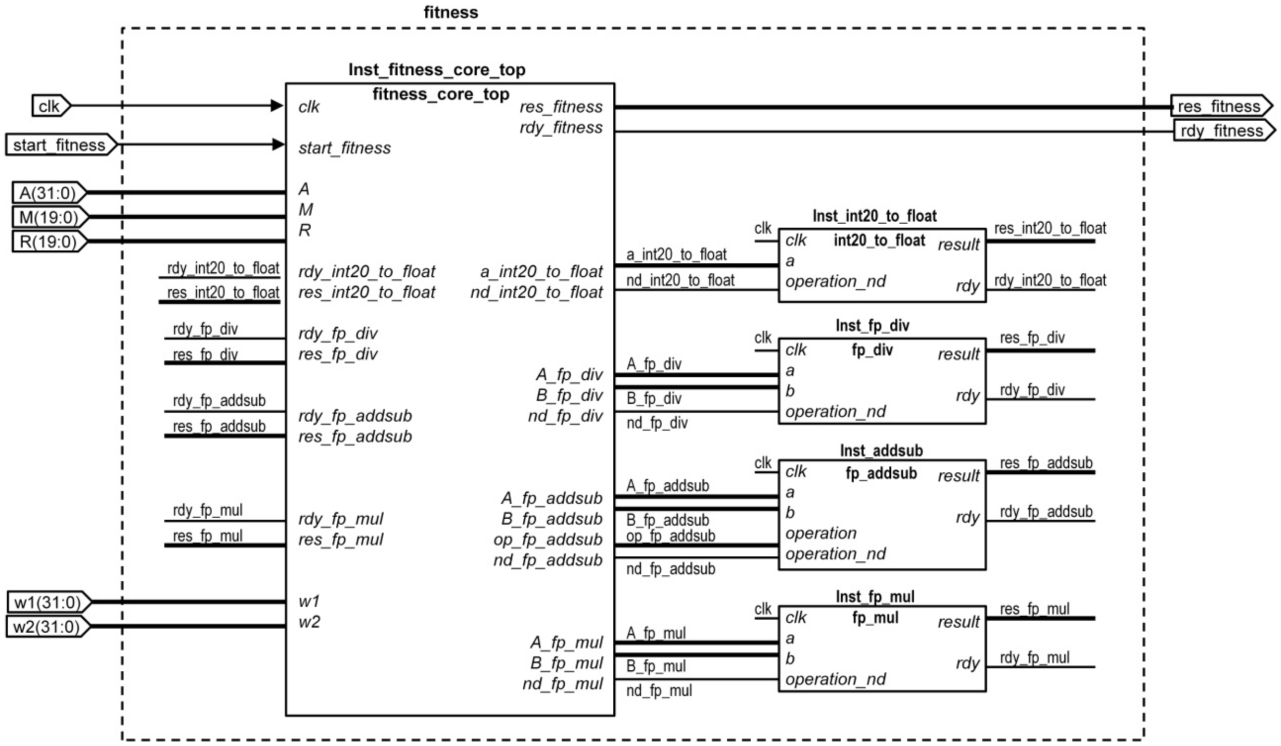

Figure S2. Top view of the fitness core.

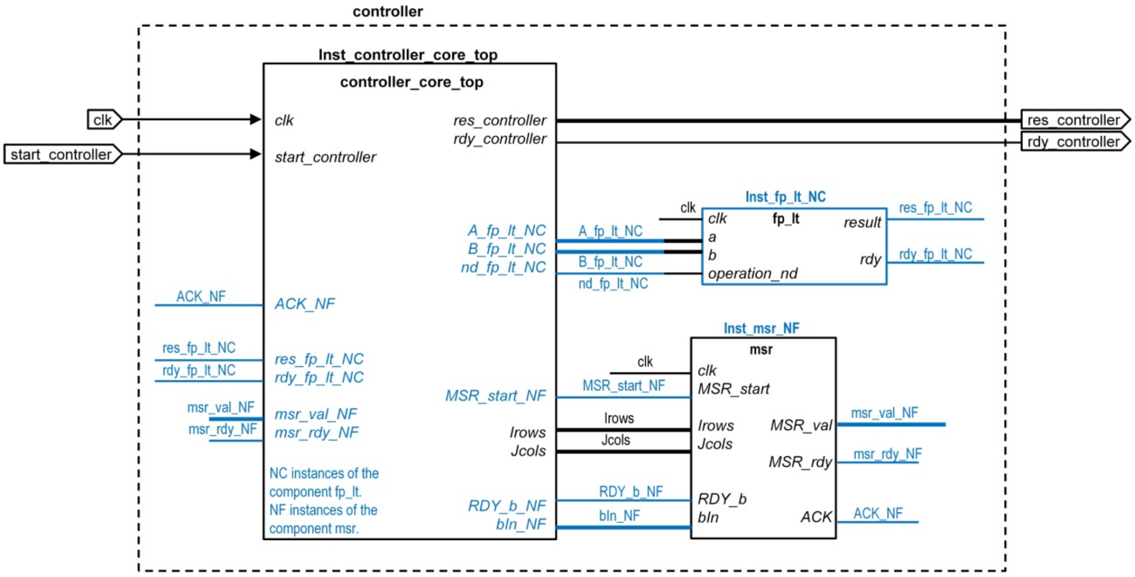

Figure S3. Top view of the MSR controller.

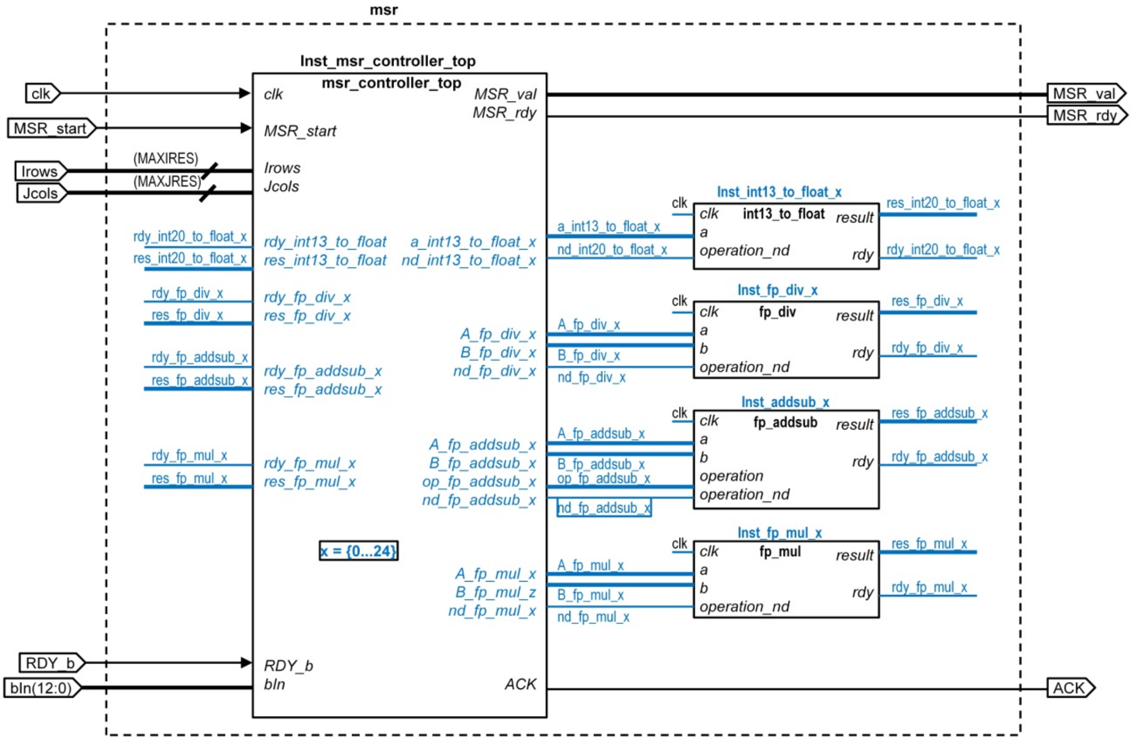

Figure S4. Top view of the MSR fitness core.
